# Sequential occurrence of microscopic polyangiitis and anti-glomerular basement membrane disease in a patient with small cell lung cancer: a case report

**DOI:** 10.1186/s13256-020-02614-3

**Published:** 2021-02-01

**Authors:** Yusuke Hayashi, Yuko Katayama, Minoru Sakuragi, Ayano Hayashi, Hiroko Kakita, Michihiro Uyama, Satoshi Marumo, Motonari Fukui

**Affiliations:** 1 Department of Respirology, Kitano Hospital, The Tazuke Kofukai Medical Research Institute, 2-4-20 Ohgimachi, Kita-ku, Osaka, 530-8480 Japan; 2 Department of Nephrology and Dialysis, Kitano Hospital, The Tazuke Kofukai Medical Research Institute, Osaka, Japan

**Keywords:** Anti-glomerular basement membrane disease, Double-positive disease, Microscopic polyangiitis, Small cell lung cancer

## Abstract

**Background:**

The association between a preceding malignancy and the onset of anti-neutrophil cytoplasmic antibody (ANCA)-associated vasculitis (AAV) has been reported in several studies. While the co-existence of ANCA and anti-glomerular basement membrane (GBM) antibodies in an individual patient is not a common occurrence, this double-positive disease currently has no optimal treatment method. Herein, we report a case of a double-positive disease involving the sequential development of acute kidney injury (AKI) and diffuse alveolar hemorrhage (DAH) in a patient with small cell lung cancer (SCLC).

**Case presentation:**

A 75-year-old Japanese woman was diagnosed with small cell lung cancer (cT3N2M1b cStage IV) and received chemotherapy. After one cycle of chemotherapy, she experienced fever and malaise. Her serum creatinine level rapidly increased, and she tested positive for myeloperoxidase (MPO)-ANCA and anti-GBM antibody. She was diagnosed with AKI due to microscopic polyangiitis (MPA) based on renal biopsy. Corticosteroid therapy was initiated, which improved her renal dysfunction. Eight days after she was discharged from the hospital, she complained of dyspnea and bloody sputum, and her condition rapidly progressed to respiratory failure. Upon chest imaging, ground-glass opacities were seen in her bilateral lower lungs. Laboratory examinations after admission revealed a lower MPO-ANCA titer and an elevated anti-GBM antibody titer compared to her previous admission. We diagnosed her with DAH due to an anti-GBM disease. After corticosteroid pulse therapy, plasma exchange was performed five times; her oxygen saturation and chest radiologic findings improved gradually. Following five cycles of plasma exchange, her oxygen saturation recovered to 95% in room air.

**Conclusions:**

To our knowledge, this is the first reported case of vasculitis caused by MPA and anti-GBM disease leading to the development of AKI and DAH during treatment of SCLC. SCLC, MPA, and anti-GBM disease may occur sequentially. A double-positive disease might have a worse prognosis; therefore, intensive therapy is more likely to achieve a better outcome.

## Background

Anti-neutrophil cytoplasmic antibody (ANCA)-associated vasculitis (AAV), including microscopic polyangiitis (MPA), comprise small- to medium-vessel vasculitides that affect multiple organs and are life-threating when untreated [1]. Anti-glomerular basement membrane (GBM) disease or Goodpasture’s syndrome is a rare small vessel vasculitis that affects the capillary beds of the kidneys and lungs. Anti-GBM disease is caused by circulating antibodies directed against an antigen intrinsic to the glomerular basement membrane [2].

Co-presentation of ANCA and anti-GBM antibodies is not uncommon. Between 7 and 41% of patients with anti-GBM disease also test positive for ANCA at the time of diagnosis, and it is more common in the elderly [3, 4]. Several studies have reported the outcomes of these “double-positive” patients, but they are still controversial; some have observed better outcomes in double-positive disease compared with single-positive anti-GBM disease, whereas others have suggested that double-positive patients have comparable or worse outcomes [5].

In some cases of AAV, malignancy may play a critical role in triggering the vasculitic process. A recent meta-analysis showed that AAV was associated with an increased risk of cancer, with a standardized incidence ratio of 1.74. However, the relationship between AAV and malignancies remains controversial [6]. Currently, the association between malignancies and anti-GBM disease is unknown. We report, to the best of our knowledge, the first case of MPA and anti-GBM disease that sequentially developed during the treatment of small cell lung cancer (SCLC). The present case raises awareness of the potential for SCLC, MPA, and anti-GBM disease to occur sequentially.

## Case presentation

A 75-year-old Japanese woman presented to another hospital with a fever in August 2018. Chest x-ray and computed tomography (CT) showed a mass in her lower right lobe. She was suspected to have a lung abscess and was treated with antibiotics, but her fever was sustained. Thus, she presented to our hospital for further investigation. Her past medical history included chronic obstructive pulmonary disease and hypertension. She had smoked approximately two packs of cigarettes a day for 53 years and had quit smoking 2 years ago. She was taking theophylline, vonoprazan, amlodipine, and tiotropium bromide hydrate/olodaterol hydrochloride. We conducted bronchoscopy and diagnosed her with SCLC (cT3N2M1b, cStage IVA according to the 8th edition of the International Union Against Cancer TNM Staging System for Lung Cancer). She was administered carboplatin and etoposide and was discharged.

**Fig. 1.**
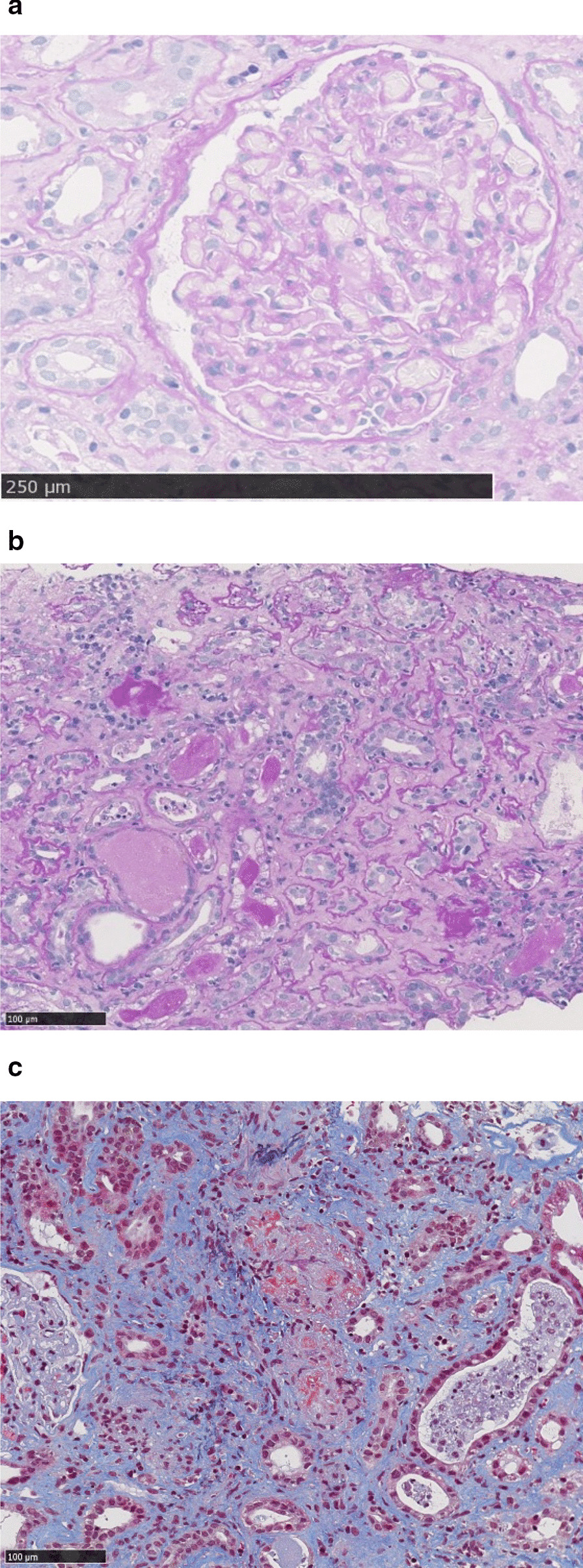
Light microscopic examinations of the renal biopsy specimens. **a** Glomerular endocapillary proliferation was observed, although there was no crescent formation (periodic acid-Schiff staining). **b **Diffuse tubular degeneration, atrophy, and diffuse cellular infiltration in the interstitium (periodic acid-Schiff staining). **c **Necrotizing vasculitis (Masson’s trichrome staining)

Four days after the discharge, she had fever and malaise; her serum C-reactive protein (CRP) was elevated at 11.4 mg/dl. She was re-admitted and treated with antibiotics. The main laboratory results from the second admission are shown in Table 1. Chest x-ray showed a regression of the mass in her right lower lobe, and there were no shadows suggesting pneumonia. After the admission, her fever still persisted, and her serum creatinine level rose rapidly from 1.06 mg/dl (day 1) to 3.51 mg/dl (day 10). Her urine test showed 1+ protein, 1+ occult blood, and urinary sediments of red blood cells. Ten days after admission, the myeloperoxidase (MPO)-ANCA titer was 82.4 U/ml (normal range, < 3.5 U/ml) and the anti-GBM antibody titer was 4.6 U/ml (normal range, < 3.0 U/ml). On day 12, her serum creatinine level increased to 4.26 mg/dl; thus, we initiated corticosteroid pulse therapy (methylprednisolone, 1000 mg/day for 2 days). On day 14, a renal biopsy was performed and revealed glomerular endocapillary proliferation, although there was no crescent formation (Fig. 1). Diffuse tubular degeneration and atrophy were observed, and cellular debris was present in the tubules. Necrotizing vasculitis and diffuse interstitial cellular infiltration were also observed. Upon immunofluorescence, no immune deposits were visible in the glomerular basement membrane and tubular basement membranes (pauci-immune). Based on these results, we diagnosed her with acute kidney injury (AKI) due to microscopic polyangiitis (MPA). After corticosteroid pulse therapy, she was administered 30 mg/day (0.5 mg/kg/day) oral prednisolone. Her fever disappeared, serum CRP and creatinine levels improved, and the MPO-ANCA titer decreased. She was then discharged again.

**Table 1:** Main laboratory results on the second admission

Hematology		
White blood cell	2300	/µl
Neutrophil count	34.6	%
Red blood cell	263	10^4^/μL
Hemoglobin	7.6	g/dl
Platelet	11.9	10^4^/μL
Blood chemistry		
Aspartate aminotransferase	13	U/l
Alanine aminotransferase	9	U/l
Lactate dehydrogenase	169	U/l
Alkaline phosphatase	243	U/l
γGTP	28	U/l
Albumin	3.7	g/dl
Blood urea nitrogen	20.1	mg/dl
Creatinine	1.06	mg/dl
C-reactive protein	11.42	mg/dl
Immunology		
Rheumatoid factor	60	
Antinuclear antibody	< 40	
PR3-ANCA	< 1.0	U/ml
MPO-ANCA	82.4	U/ml
Anti-GBM antibody	4.6	U/ml
Tumor marker		
ProGRP	1520	pg/ml
Neuron-specific enolase	11	ng/ml
Urinalysis		
Protein	1+	
Protein excretion	1091.5	mg/gCr
Sugar	-	
Occult blood	±	
NAG	18.8	IU/l
β2MG	1619	µg/l

*GBM* glomerular basement membrane, *MPO-ANCA* myeloperoxidase-anti-neutrophil cytoplasmic antibodies, *NAG N*-acetyl glucosaminidase, *ProGRP* progastrin releasing peptide, *PR3-ANCA* proteinase-3-anti-neutrophil cytoplasmic antibodies, *γGTP* gamma-glutamyl transpeptidase, *β2MG* β 2-microglobulin

**Fig. 2.**
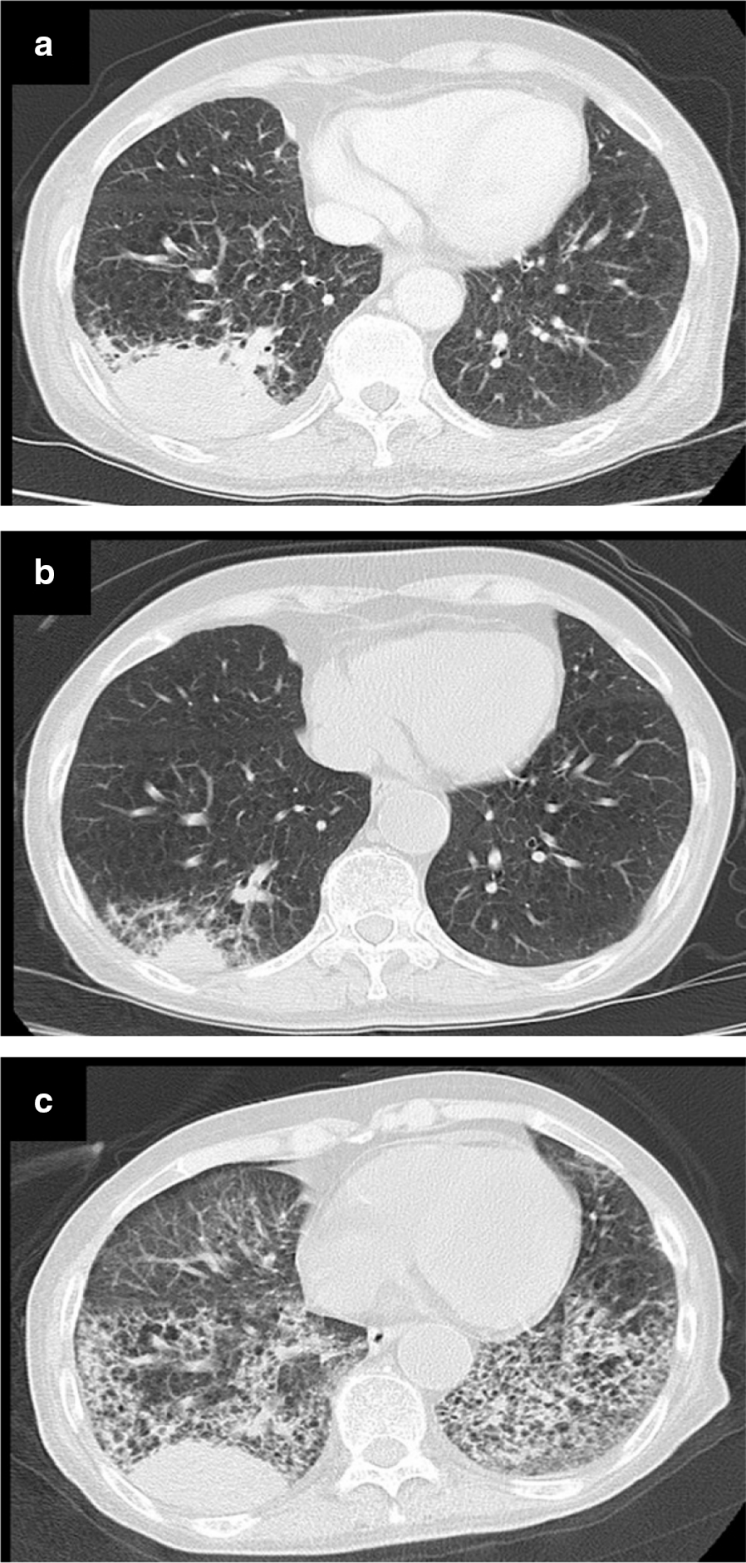
Time course of chest computed tomography. **a** First admission on August 2018. A large mass was observed in the lower lobe of the right lung. **b** Second admission on September 2018. Chemotherapy resulted in shrinking of the lung mass. **c** Third admission on October 2018. Ground-glass opacities in the bilateral lower lung fields. The masses are re-growing

Only 7 days after the second discharge, the patient complained of dyspnea and returned to our emergency department. On physical examination, her blood pressure was 146/84 mmHg; she had a pulse rate of 108 beats per minute, respiratory rate of 24 breaths per minute, and oxygen saturation of 85% on 10 l/min of oxygen via non-rebreather mask. On chest auscultation, coarse crackles were bilaterally detected in the lower back. Chest x-ray and CT showed ground-glass opacities in the bilateral lower lung fields (Fig. 2). After admission, the patient frequently produced bloody sputum. Although we could not perform bronchoscopy because of her deteriorated respiratory state, we made a clinical diagnosis of diffuse alveolar hemorrhage (DAH) and started corticosteroid pulse therapy again. After corticosteroid pulse therapy, her oxygen demand decreased from 10 l/min to 5 l/min. Dyspnea and bloody sputum also partially improved. Four days after the third admission, laboratory investigations revealed that the MPO-ANCA titer decreased to 13.2 U/ml, but the anti-GBM antibody titer increased to 43.3 U/ml. Since the serum creatinine level remained at 2.12 mg/dl after the second discharge, there was no need to perform renal biopsy again. Given that the MPO-ANCA titer was lower and the anti-GBM titer was higher than those previously determined at the time of her second admission, we speculated that the DAH was probably a concurrence with the anti-GBM disease rather than an aggravation of MPA. Therefore, we performed plasma exchanges (PEs) following corticosteroid pulse therapy. After five cycles of PE, her oxygen saturation increased to 95% in room air, with improved chest radiographic findings.

**Fig. 3.**
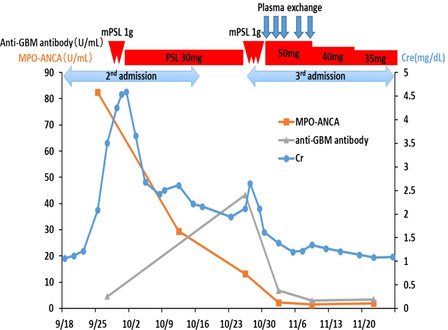
The clinical course of the patient. Abbreviations Cr: creatinine, GBM: glomerular basement membrane, MPO-ANCA: myeloperoxidase-anti-neutrophil cytoplasmic antibody, mPSL; methylprednisolone

During treatment for AKI and DAH, we could not administer chemotherapy for SCLC; this resulted in a gradual growth of her tumor. Since her activities of daily living and performance status had deteriorated because of frequent hospitalizations, we could not restart chemotherapy. The treatment course of this patient is shown in Fig. 3.

## Discussion and conclusions

To the best of our knowledge, this is the first reported case of a patient with SCLC complicated by the sequential development of MPA and anti-GBM disease. This patient responded well to intensive treatment with corticosteroid therapy plus PE.

When AKI occurred during the second admission, the coexistence of ANCA and anti-GBM antibody was recognized in the patient. Based on the renal biopsy, we diagnosed AKI due to MPA rather than due to anti-GBM disease. Her renal dysfunction improved with the administration of corticosteroid pulse therapy followed by daily 30-mg oral prednisolone.

However, the patient went on to develop rapid-onset DAH, despite the ongoing administration of prednisolone. Although it is possible for DAH to be affected by the exacerbation of MPA, her MPO-ANCA titer was lower than that determined during her second admission, while her anti-GBM antibody titer was elevated. Some studies have shown that ANCA and anti-GBM antibody titers are associated with disease activity and relapse [7, 8]. Thus, these changes in ANCA and anti-GBM antibody titers in our patient indicated that DAH could be associated with anti-GBM disease rather than an aggravation of MPA. The effectiveness of PE in addition to corticosteroid pulse therapy further supported this diagnosis.

The concurrence of ANCA and anti-GBM antibodies as in the present case is well documented. A review of these double-positive patients reported that 5% of ANCA-positive patients tested positive for anti-GBM antibody, whereas 32% of anti-GBM antibody-positive cases were also positive for ANCA [9, 10].

Low levels of ANCA may be observed years before anti-GBM antibodies become detectable [9]. Although the specific contribution of ANCA to the mechanism of anti-GBM disease is largely unknown, one hypothesis is as follows: when activated by ANCA, neutrophils are postulated to release reactive oxygen species and lytic toxic granule enzymes, causing injury to the surrounding tissues. In this process, the GBM epitope is exposed, initiating the development of antibodies against GBM [11–13]. In fact, previous case reports have noted that, similar to this case, MPA and anti-GBM disease may develop sequentially [14].

Double-positive disease has similar characteristics to AAV, including age distribution, duration of symptoms, and tendency to relapse. However, the initial clinical presentation of double-positive disease is more in line with anti-GBM disease, including a high frequency of dialysis-requiring kidney failure and alveolar hemorrhage [5]. McAdoo *et al.* reported that double-positive patients experience the early morbidity and mortality typical of anti-GBM disease, and they carry the long-term risk of relapse typical of AAV [5].

There are no randomized-controlled trials investigating whether double-positive disease should be treated with immunosuppressive drugs alone or with a combined therapy of immunosuppressive drugs and PE. A literature review reported the outcomes of 52 double-positive patients and showed that the survival rate of patients who received both immunosuppressive drugs and PE is 74% (26 of 35 patients), whereas the survival rate with immunosuppressive drugs alone is only 53% (9 of 17 patients) [15].

In the present case, the patient was positive for both MPO-ANCA and anti-GBM antibody at the time of AKI diagnosis. Since immunofluorescence of the kidney biopsy demonstrated no linear deposition of IgG along the glomerular capillaries, we diagnosed the patient with MPA alone rather than complicated by anti-GBM disease and initiated only corticosteroid therapy. Although the renal dysfunction and high MPO-ANCA titer immediately improved, DAH sequentially occurred in parallel with an increased titer of anti-GBM antibody. Taking into account this clinical course and the double-positive disease, we should have initiated treatment with both immunosuppressive drugs and PE at the time of AKI diagnosis.

Our patient sequentially developed MPA and anti-GBM disease after the diagnosis of SCLC. At the time of her SCLC diagnosis, the MPO-ANCA and anti-GBM antibody titers were not measured, as there were no clinical findings suggestive of MPA or anti-GBM disease. The relationship between vasculitis and malignancy remains unclear and controversial. The association between a preceding malignancy and the onset of AAV has been investigated in several studies. A retrospective study showed that the overall risk for malignancy prior to AAV diagnosis was similar to that of the general population [16]. On the other hand, Pankhurst *et al.* reported that AAV increased the risk of concurrent or preceding malignancy [17]. The increased risk of malignancy in patients with AAV has been largely attributed to the carcinogenic effects of cyclophosphamide [18].

Some pathogenic mechanisms have been postulated to explain the association between vasculitis and malignancy. Tumor cells may cause immunologic reactions against the vascular endothelium, release various cytokines causing endothelial injury, induce delayed hypersensitivity reaction by the deposition of tumor proteins on vessel walls, produce critical levels of circulating immune complexes that injure the endothelial cells of post-capillary venules, and so on. [19].

According to our research, there are no previous reports of SCLC preceding AAV or anti-GBM disease. Additionally, the association between malignancy and anti-GBM disease has never been reported. Lung cancer, especially SCLC, is sometimes associated with paraneoplastic syndromes. The proposed mechanisms of paraneoplastic processes include the aberrant release of humoral mediators from tumor cells, such as hormones and hormone-like peptides, cytokines, and antibodies [20]. In this case, SCLC might trigger MPO-ANCA production. Although we cannot deny the possibility that these diseases might happen coincidentally, SCLC might trigger an occurrence of MPA followed by anti-GBM disease in some pathogenic mechanisms.

In conclusion, we present a rare case of a double-positive disease involving the sequential development of AKI and DAH in a patient with SCLC. The key message from this case report is that lung cancer, MPA, and anti-GBM disease might be closely associated in terms of their pathogenesis and manifestation. Additionally, patients with double-positive diseases may develop AKI and DAH more frequently (like anti-GBM disease) rather than AAV alone. The administration of immunosuppressive drugs plus PE at the time of diagnosis of double-positive disease may lead to better outcomes. Further studies are needed to validate these speculations.

## Data Availability

The datasets used during the current study are available from the corresponding author on reasonable request.

## Abbreviations

AAV: Anti-neutrophil cytoplasmic antibody-associated vasculitis
AKI: Acute kidney injury
ANCA: Anti-neutrophil cytoplasmic antibody
CRP: C-reactive protein
CT: Computed tomography
DAH: Diffuse alveolar hemorrhage
GBM: Glomerular basement membrane
MPA: Microscopic polyangiitis
MPO: Myeloperoxidase
PE: Plasma exchange
SCLC: Small cell lung cancer
